# The effectiveness of esomeprazole 40 mg in patients with persistent symptoms of gastro-oesophageal reflux disease following treatment with a full dose proton pump inhibitor

**DOI:** 10.1111/j.1742-1241.2008.01923.x

**Published:** 2008-12

**Authors:** R Jones, T Patrikios

**Affiliations:** 1 Department of General Practice & Primary Care, King’s College LondonLondon, UK; 2 AstraZeneca UKLuton, UK

## Abstract

**Background:**

Some patients with gastro-oesophageal reflux disease (GORD) remain symptomatic despite proton pump inhibitor (PPI) treatment. There is a need to determine the most appropriate management of these patients.

**Aims:**

To assess the effectiveness of esomeprazole 40 mg in GORD symptoms persisting in patients receiving a full daily dose PPI.

**Methods:**

In this multi-centre open label study patients who had received full daily dose PPI for 8 weeks, but were still experiencing persistent GORD symptoms, were treated with esomeprazole 40 mg for 8 weeks (*n* = 99). The primary outcome variable was the change in the frequency of heartburn. Patient-reported outcomes were also assessed using the Reflux Disease Questionnaire (RDQ) and the GORD Impact Scale (GIS).

**Results:**

The mean frequency of heartburn was reduced by 78% from 4.4 days a week to 1 day a week at the end of the 8-week treatment period (p < 0.0001). Other GORD symptoms were also significantly reduced following of treatment with esomeprazole (all p < 0.0001). All RDQ dimensions and the level of symptom control as measured by the GIS also showed significant improvement at 8 weeks.

**Conclusions:**

In patients with persistent GORD symptoms despite full dose daily PPI therapy, esomeprazole 40 mg significantly improved the frequency and severity of all GORD symptoms.

**What's known**

A significant minority of patients treated for gastro-oesophageal reflux disease (GORD) remain symptomatic despite therapy with proton pump inhibitor agents. The reasons for this are not clear, nor are the best approach to management. More effective acid suppression is one possible management strategy.

**What's new**

Treatment with esomeprazole 40 mg daily in patients with GORD who have responded incompletely to a full daily dose of other PPIs is associated with a significant reduction in symptoms as measured with the Reflux Disease Questionnaire and an improvement in quality of life, measured with Gastro-oesophageal Disease Impact Scale.

## Introduction

Gastro-oesophageal reflux disease (GORD) is a common disorder, with 10–20% of the population estimated to have the condition and consultations for dyspepsia accounting for 1.2–4% of all primary care consultations in the UK (1). The cardinal symptoms of GORD, heartburn and acid regurgitation, are often accompanied by other symptoms, which together have a substantial negative impact on patients’ quality of life (2,3). Despite the efficacy of proton pump inhibitor (PPI) therapy, a number of studies have indicated that a significant minority of GORD patients (20–25%) (4) receiving PPIs continue to have unresolved symptoms, accompanied by continuing impairment of their quality of life (5,6).

The National Institute for Health and Clinical Excellence recommends that general practitioners (GPs) adopt a symptom-driven approach to patient management (7), which is in keeping with the observation that symptoms, rather than endoscopic appearances, are a better guide for assessing the response to therapy. Jones et al. (8) have recently shown that patients who respond best to therapy subsequently enjoy the best quality of life.

Primary care physicians continue to experience problems in managing GORD, and there is evidence that patients and clinicians perceive the severity and impact of symptoms differently (9). To improve the ascertainment of patients’ symptoms, a number of patient questionnaires have been developed, most recently the GIS (GORD Impact Scale), which has been extensively validated in the primary care setting and shown to be responsive to changes in patients’ symptoms (10).

In the face of persistent GORD symptoms, when attention has been paid to lifestyle factors and an explanation about mechanisms and the effects of therapy has been provided, clinicians are faced with the dilemma of the appropriate next therapeutic step, which could be to refer for a specialist opinion, to arrange an endoscopy or to change the medication. Esomeprazole has been shown to provide better acid-control than other PPIs (11–13), and is also more effective at healing oesophagitis (14–16). This study was undertaken to assess whether esomeprazole 40 mg is effective when other PPIs, prescribed at a full daily dose for a period of up to 8 weeks, have failed to adequately control the symptoms of GORD.

## Methods

### Study design

We undertook a multi-centre open label study in the UK, in which patients were treated with esomeprazole 40 mg for 8 weeks. Patients attended a screening visit before initiation of study treatment (baseline) followed by two additional clinic visits at 4 and 8 weeks.

### Patients

Patients who were followed up by their GP for GORD treatment were invited to participate in the study when it had become apparent that they were still experiencing symptoms of GORD, defined as heartburn, epigastric pain or acid regurgitation, despite being on their current PPI therapy. This is to reflect how patients are currently managed in primary care. Informed consent was obtained at the screening visit and patients eligible to enter the study were issued with study medication. Patients’ symptom status was reassessed after 4 weeks of treatment and at the third visit at 8 weeks. Patients eligible for inclusion in the study were men and women aged over 18 who were currently being treated for GORD with a PPI at full daily dose (i.e. omeprazole 20 mg daily, lansoprazole 30 mg daily, rabeprazole 40 mg daily) for a preceding period of up to 8 weeks. In order to be eligible, patients were required to report persisting GORD symptoms of heartburn, acid regurgitation or epigastric pain during the 7 days prior to the first visit, either as 4 days with mild symptoms or 2 days with moderate to severe symptoms (see Table 1).

**Table 1:** Symptom severity rating

Mild (1)	Awareness of symptom(s) which is/are easily tolerated
Moderate (2)	Discomfort sufficient to cause interference with normal activities
Severe (3)	Incapacitating with inability to perform normal activities

Patients were excluded from the trial if they had had more than one previous course of full dose PPI in the last year or had been treated for more than 8 weeks with their current full dose PPI. Patients who had previously taken esomeprazole or those who were using an H2 receptor antagonist were also excluded. Patients with serious gastrointestinal, cardiovascular, metabolic or other conditions were excluded, as were those with a history of upper gastrointestinal surgery. Patients with irritable bowel syndrome, with any evidence of gastrointestinal alarm symptoms or severe concurrent disease or mental illness, were also excluded. Continuous concurrent therapy with a range of potentially interactive drugs was also an exclusion criterion, as were pregnancy and lactation, chronic alcoholism, current malignancy, known hypersensitivity to esomeprazole or participation in a clinical study during the last 90 days.

### Efficacy assessment

The primary outcome of the study was an assessment of the change in the frequency of heartburn from baseline to the end of the study at 8 weeks. The secondary outcome measures included assessments of the change of frequency of heartburn after 4 weeks of treatment, and the change in severity and frequency of heartburn, epigastric pain and acid regurgitation at 4 and 8 weeks.

At each visit, patients were questioned in a standardised manner by the investigator about the frequency of their GORD symptoms. The overall severity of each symptom was measured on a 4-point scale from zero (none) to three (severe) (see Table 1). Patients were also asked how much relief they obtained from their over-the-counter (OTC) medication, during the previous 12 months.

Other patient-reported outcome measures were changes in symptom control from baseline to 8 weeks as assessed by the RDQ (Reflux Disease Questionnaire) and the GIS. The RDQ is a 12-item self-administered questionnaire design to assess the frequency and severity of heartburn, acid regurgitation and epigastric pain (17). Symptom frequency and severity are scored on a 6-point Likert scale. Twelve items are combined into three dimensions: heartburn, regurgitation, dyspepsia. The mean of all three dimensions gives a total score. The specific GORD dimension is determined by the mean of the dimensions of heartburn and regurgitation. Physicians’ assessments of GORD symptoms were correlated with the RDQ scores.

The GIS is a reliable tool, validated for use in clinical practice (10). It consists of nine questions, which ask about the frequency of a range of symptoms, including sleep, eating and daily activities, as well as the cardinal symptoms of reflux disease, each scored on a 4-point scale from ‘never’ to ‘daily’. The level of symptom control for individual patients is determined by the number of ticks in each of the four parameters (never-daily). Nine ticks in the ‘never’ box signifies that GORD symptoms are well-controlled; six ticks in the ‘never’ box indicates ‘fairly well-controlled’ GORD symptoms; > 3 ticks outside the ‘never’ box signifies uncontrolled GORD; > 5 ticks outside the ‘never’ box signifies poorly controlled; and all nine ticks outside the ‘never’ box signifies ‘very poorly controlled’. This outcome variable reflects how physicians currently use the GIS in determining how well-controlled the GORD symptoms are in a patient.

### Tolerability and safety assessment

The nature, incidence and severity of serious adverse events (SAEs) and adverse events leading to discontinuation of a patient from study treatment were analysed for all patients who took at least one dose of the investigational product and for whom postdose information was available.

### Statistical analysis

The primary and secondary efficacy and patient-reported outcome variables were analysed using a full analysis set, which included all patients with at least one assessment of efficacy after initiation of study treatment. The safety variables were analysed using the safety analysis set, which consisted of all patients who took at least one dose of the investigational product, and for whom postdose information was available. For the primary and secondary efficacy variables, the changes from baseline to 4 and 8 weeks scores in the frequency and severity of the GORD symptoms of heartburn, acid regurgitation and epigastric pain were analysed by the Wilcoxon signed rank test.

Secondary patient-reported outcome variables, including the changes from baseline to week 4 and week 8 scores on the RDQ global and dimension scores were analysed by the Wilcoxon signed rank test. The GIS data were summarised using descriptive statistics. The correlation between the RDQ domain scores and the physicians’ assessment of these symptoms were analysed using the Spearman rank correlation coefficient. Statistical analyses were performed by using the sas system (SAS version 8.2, SAS Institute Inc., Cary, NC).

### Sample size

The sample size calculation was based on an estimation of the change in frequency of heartburn from baseline to 8 weeks. To detect a reduction (treatment effect) of at least 1 day in the frequency of heartburn during the previous week, assuming a standard deviation of 2.8 and at the 5% significance level with 90% power, 85 patients were required in the study. We assumed a dropout rate of 15% and therefore planned to enrol approximately 100 patients.

## Results

In total, 99 patients from 17 research sites in the UK were enrolled in the study. All patients entering the study were analysed for safety and 94 (95%) were analysed for efficacy and patient-reported outcomes (five patients were excluded from the full analysis set because no assessment of efficacy was made after baseline data were collected).

The demographic features and current treatment of these patients are shown in Table 2. The mean age of the population was 46 years and all were of Caucasian origin. Reflux symptoms had been present for a mean of 4.5 years and heartburn had been experienced for a mean of 4.4 days during the week before the trial. 63% of the patients had little or no relief of symptoms when using OTC preparations in the last 12 months in addition to taking their current PPI.

**Table 2:** Baseline characteristics and treatment of patients included in the efficacy analysis

Demographic characteristic	*n* = 94
**Gender (*n* and % of patients)**	
Male	37 (39.4%)
Female	57 (60.6%)
**Age (years)**	
Mean (SD)	46.1 (16.1)
**Race (*n* and % of patients)**	
Caucasian	94 (100.0%)
**History of GORD symptoms (months)**	
Mean (SD)	56.1 (97.9)
**Duration of current episode of GORD (weeks)**	
Mean (SD)	9.53 (8.78)
**Frequency of heartburn in last week (days)**	
Mean (SD)	4.4 (2.3)
**Previous PPI (*n* and % of patients)**	
Lansoprazole 30 mg	25 (26.6%)
Omeprazole 20 mg	67 (71.3%)
Rabeprazole 40 mg	2 (2.1%)

GORD, gastro-oesophageal reflux disease; PPI, proton pump inhibitor.

**Figure 1:**
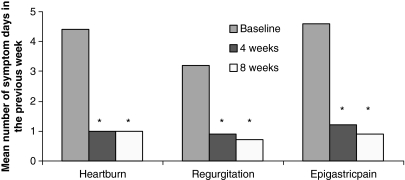
Change in frequency of symptoms (*p < 0.0001; *n* = 94)

**Figure 2:**
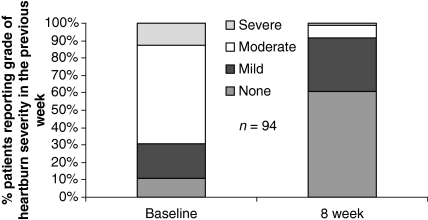
Change in the grade of heartburn severity (*n* = 94)

Changes in the primary and secondary outcome variables are summarised in Figures 1 and 2.

The mean frequency of heartburn was reduced from 4.4 days a week to 1 day a week at the end of the 8-week treatment period with esomeprazole 40 mg p < 0.0001 (95% CI: –4.0, −2.8). This corresponds to a mean reduction of 78% in the frequency of heartburn.

Control of heartburn symptoms was achieved within 4 weeks of starting treatment with esomeprazole with a mean reduction in the frequency of heartburn to 1 day a week (mean change in frequency of heartburn from baseline −3.4 days, p < 0.0001) Frequency of acid regurgitation was significantly reduced by 2.6 days per week at 8 weeks (95% CI: −3.1, −2.1; p < 0.0001) as was the frequency of epigastric pain by 3.6 days per week after 8 weeks of treatment with esomeprazole (95% CI: −4.2, −3.0; p < 0.0001). The benefit became apparent as early as 4 weeks after initiation of treatment with esomeprazole 40 mg. This was also accompanied by a significant reduction in the severity of heartburn from baseline to 8 weeks (Figure 2) as well as the severity of acid regurgitation and epigastric pain with esomeprazole (all p < 0.0001). At all time points, there was a substantial and statistically significant reduction in GORD symptom frequency and severity.

**Figure 3:**
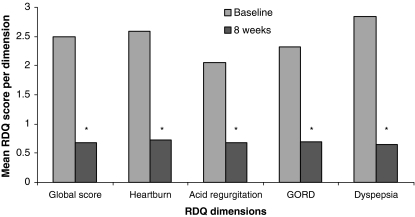
Change in Reflux Disease Questionnaire scores. (*p < 0.001; *n* = 94)

These changes are reflected in improvements in the RDQ global score and in the heartburn, acid regurgitation, dyspepsia and GORD dimension scores of the RDQ at 8 weeks of esomeprazole 40 mg treatment (Figure 3). Similar results were observed after 4 weeks of treatment.

The RDQ scores and physicians’ assessment were also well correlated. Changes in the RDQ scores (heartburn, acid regurgitation, epigastric pain) and changes in physician’s assessment of these symptoms were well correlated (Spearman’s rank correlation coefficient 0.80, 0.83 and 0.69 respectively).

**Figure 4:**
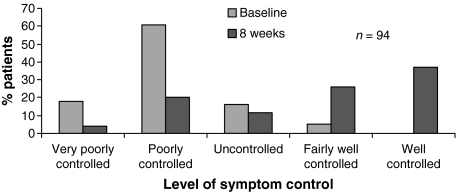
Change in the level of control assessed by the Gastro-oesophageal Reflux Disease Impact Scale (*n* = 94)

The GIS scores also showed a considerable improvement at 4 and 8 weeks and the level of symptom control, assessed by summarising the scores on the GIS, is shown in (Figure 4) emphasising that patients gradually moved from poorly controlled to well-controlled over the 8 week study period.

### Safety

The mean duration of exposure to esomeprazole 40 mg was 51 days and, overall, the drug was well-tolerated. There were three SAEs: admission for myocardial infarction, chronic obstructive pulmonary disease (COPD) exacerbation and balance disorder. None of the SAEs were considered causally related to the study drug. Four of the adverse events led to discontinuation – diarrhoea, abdominal distension, flatulence, exacerbation of GORD – and were assessed as related to esomeprazole treatment. Diarrhoea was the only adverse event reported by more than one patient.

## Discussion

This study shows that patients who continue to experience GORD symptoms on full dose PPI therapy obtain substantially improved symptom control when treated with esomeprazole 40 mg daily. We have shown improvements, which are statistically significant and considered to be clinically important, in a range of patient-reported outcome measures, including the RDQ and the GIS.

The persistence of reflux symptoms in patients taking PPIs is now a well-recognised problem. Although we do not have a measure of compliance in our patients, non-response has been observed in fully compliant subjects. This has a number of implications for clinicians. The first of these, of course, is to ensure that patients are assessed adequately and the introduction of the GIS, designed to support clinicians’ questioning patients about their symptom status, is one potentially valuable approach to ensuing that this takes place. When appropriately questioned, as many as 25% of patients on PPIs report persistent symptoms (4), and these are associated with substantial impairment of quality of life (2,3). Confronted with a ‘non-responder’ to full-dose PPI therapy, the primary care clinician has a number of options, which include specialist referral and investigation with upper gastrointestinal endoscopy and possibly pH monitoring and manometry. Another option is to prescribe a double dose of the current PPI, in order to achieve better acid suppression and consequent symptomatic improvement. However, there are data to suggest that this may not be a clinically effective approach. Rohss et al. (11) showed that esomeprazole maintains intragastric pH above 4 for a longer period of time than omeprazole 40 mg, equating to an additional 1.5–2 h of acid suppression. There is also evidence from the same study that interpatient variability in the percentage of the time for which gastric pH exceeds 4 is significantly less with esomeprazole 40 mg than with omeprazole 40 mg daily. The study by Wilder-Smith et al. (12) compared esomeprazole 40 mg daily with lansoprazole 60 mg daily (i.e. double the normal daily dose) and found that acid control, in terms of the duration of time during which intragastric pH exceeded 4, was significantly better with esomeprazole than with high-dose lansoprazole. Finally, a recent meta-analysis (18), conducted as part of a Cochrane systematic review, and which examined 18 randomised controlled trials comparing standard dose and double dose PPI, was unable to identify data to support the practice of doubling the dose of PPI therapy in patients who do not respond to standard dose therapy, with the possible exception of esomeprazole 40 mg once daily.

These choices have significant resource implications for health services, both in terms of the costs of investigations, repeated visits to the GP and the costs of drugs. In some healthcare systems, a double dose of a standard PPI may be more expensive than a standard dose of esomeprazole. The availability of more effective acid suppression, through the use of esomeprazole 40 mg daily, offers a more rational and potentially more cost-effective approach as it rapidly controls GORD patients’ symptoms thereby reducing the need for repeat GP visits and referral for endoscopy.

The strengths of this study include its pragmatic approach, in which events in real clinical practice were captured and clinicians were asked to initiate esomeprazole 40 mg daily in patients whose GORD symptoms were not adequately controlled on their current PPI. These patients were being cared for in primary care, by GPs, and the study replicates a plausible sequence of events that does not involve specialist referral or investigation.

A significant weakness of the study, of course, is that we did not adopt a randomised design, by allocating patients to either esomeprazole or to a comparator drug (or to placebo). We considered that an open-label approach was a more appropriate design for this study, in which a placebo comparison would have been inappropriate (because of persisting symptoms), and continuation with a less effective agent also raises design and ethical questions. In this study, the patients acted as their own controls although, of course, we cannot exclude the additional benefit experienced by patients simply by virtue of being part of a clinical trial. The use of epigastric pain as an inclusion criterion and the exclusion of patients with irritable bowel syndrome may limit the generalisability of our findings, but we set out to study a patient population likely to be representative of patients with predominant GORD in primary care.

Overall, the results of the study indicated that patients whose GORD symptoms remain uncontrolled despite receiving a full dose of a PPI benefit from a change to esomeprazole 40 mg, with significant reduction in the frequency and severity of the three predominant GORD symptoms within 4–8 weeks. We think that this approach should be adopted before clinicians consider specialist intervention or endoscopic investigation, in patients without alarm symptoms or other concerns. This study’s findings also demonstrated the value of using patient-reported questionnaires as a tool in disease management. The RDQ and the GIS were easy to use, responsive and performed well in this study and the GIS, in particular, has a potentially valuable part to play in routine management of patients with GORD.
